# Changes in the microRNA cargo of granulosa cell-derived extracellular vesicles under oxidative stress in a bovine model

**DOI:** 10.1186/s40659-026-00689-8

**Published:** 2026-03-25

**Authors:** Judith Diaz-Muñoz, Yulia N. Cajas, Karina Cañón-Beltrán, Lena Arévalo, Sonia Gago, María Iniesta-Cuerda, Ana Josefa Soler, Dimitrios Rizos, Teresa Mogas

**Affiliations:** 1 Department of Animal Medicine and Surgery, Autonomous University of Barcelona, 08193 Cerdanyola del Vallès, Spain; 2 Department of Agrarian Production, Technical University of Madrid (UPM), 28040 Madrid, Spain; 3 Department of Biological Science, Technical University of Loja (UTPL), 1101608 Loja, Ecuador; 4 Escuela de Ciencias Agrícolas y Ambientales, Pontificia Universidad Católica del Ecuador- Sede Ibarra, Ibarra, Ecuador; 5 Department of Animal Reproduction, National Institute for Agriculture and Food Research and Technology (INIA-CSIC), Cta de La Coruña Km 5.9, 28040 Madrid, Spain; 6 Grupo SaBio (CSIC-UCLM-JCCM), ETSIAMB, Campus Univesitario s/n, 02071 Albacete, Spain

**Keywords:** Exosomes, Cattle, Reproductive stress

## Abstract

**Background:**

Granulosa cells (GCs) play a central role in oocyte maturation and follicular development, and their function is very sensitive to oxidative stress, which can compromise female fertility. Extracellular vesicles (EVs) released by GCs serve as intercellular messengers and are known to carry bioactive molecules, including microRNAs (miRNAs), which can modulate stress responses and cellular communication within the follicular environment. Understanding the molecular mechanisms underlying oxidative stress responses in reproductive tissues is crucial for improving fertility. This study aimed to characterize and compare the miRNA profiles of both GCs and their secreted EVs following exposure to oxidative stress. Bovine GCs were treated with 5 µM H_2_O_2_ for 40 min, followed by 24 h of culture. EVs were isolated from the conditioned media using size-exclusion chromatography and characterized by nanoparticle tracking analysis, transmission electron microscopy, and flow cytometry. Small RNA sequencing was performed on both the GCs and their secreted EVs to identify stress-responsive miRNA signatures.

**Results:**

Exposure to oxidative stress increased intracellular ROS levels and decreased mitochondrial activity in GCs, confirming successful stress induction. Differential expression analysis revealed no significant changes in miRNA abundance in GCs in response to oxidative stress, suggesting cellular adaptation mechanisms that preserve intracellular miRNA homeostasis. In GC derived EVs, however, 10 miRNAs were significantly more abundant, including miR-134, miR-10175-5p, miR-197, miR-2284 h-5p, miR-2284y, miR-2285av, miR-2285au, miR-369-5p, miR-2411-5p and miR-2387. These miRNAs showed log2 fold changes ranging from 3.57 to 6.89 compared to control, indicating substantial enrichment in stress-derived EVs. Functional enrichment analysis of predicted targets for the higher abundant EV-miRNAs revealed involvement in processes such as angiogenesis, cell migration as well as WNT, MAPK and Oxytocin signaling pathways, all critical for follicular function and stress adaptation.

**Conclusions:**

These findings suggest that oxidative stress alters the miRNA cargo of EVs, potentially affecting intra-follicular communication and stress adaptation The selective packaging of specific miRNAs into EVs under stress conditions indicates a sophisticated cellular mechanism for intercellular communication during adverse conditions. This dual characterization provides new insights into the regulatory role of EV-derived miRNAs in ovarian physiology under oxidative conditions and establishes the foundation for developing EVs-miRNAs as potential biomarkers for reproductive health assessment.

**Supplementary Information:**

The online version contains supplementary material available at 10.1186/s40659-026-00689-8.

## Introduction

Granulosa cells (GCs) are essential for ovarian follicle development, providing structural support, regulating oocyte maturation, and facilitating the exchange of nutrients and signals necessary for proper folliculogenesis​ through intercellular communication mediated by paracrine, autocrine and endocrine signaling [1]. Oxidative stress causes disruption of some signaling pathways in GCs that control cell proliferation, autophagy, and apoptosis, which can induce follicular atresia [2, 3]. In addition, this process is accompanied by other alterations, such as decreased antioxidant levels, impaired steroid synthesis, and mitochondrial dysfunction. Oxidative stress in GCs arises from various environmental and physiological challenges, including heat stress or metabolic stress. These stressors disrupt redox homeostasis by promoting excessive reactive oxygen species (ROS) production, leading to cellular damage and impaired follicular function. Although the exact mechanisms remain to be fully elucidated, several signaling pathways have been implicated in the GCs oxidative response, including PI3K/AKT, MAPK, FOXO, Nrf2/KEAP1, inflammation-related pathways, and mitophagy [2].

Oxidative stress arises from an imbalance between the production of ROS and the cellular antioxidant defense mechanisms, resulting in molecular damage and activation of stress response pathways [4]. Reactive oxygen species are free radicals, including superoxide anion (O_2_–), hydrogen peroxide (H_2_O_2_), and hydroxyl radicals (–OH), derived from intracellular metabolism or environmental stimuli and mostly produced by the mitochondria. Under physiological conditions, ROS are essential to maintain physiological processes, which include nuclear maturation of oocytes and intracellular signaling in follicular cells. During follicular development, the metabolic activity of follicular cells increases to meet the increasing demand for nutrients and energy, which naturally elevates ROS production [5].

Oxidative stress not only alters the intracellular environment but also influences the secretion and molecular composition of extracellular vesicles (EVs). EVs are particles enclosed in a lipid bilayer that carry different cytosolic macromolecules (such as mRNA, miRNAs and proteins) which can be transferred to recipient cells and induced alterations in their physiological functions [6]. Under cell stress conditions, these EVs contain different bioactive molecules-including stress-responsive miRNAs and antioxidant transcripts-that are reflective of the original cell’s physiological status and can modulate the behavior of recipient cells within the follicle [7, 8]. Using an in vitro model, Gebremedhn et al. [9] showed that bovine GCs exposed to heat stress (42 °C) exhibited increased levels of ROS, protein oxidation, apoptosis, and elevated expression of HSPs and antioxidants, along with reduced cell viability. Notably, heat stress altered the miRNA cargo with 14 and 6 miRNAs differentially expressed in heat-stressed GC and their corresponding EVs, suggesting an EV-mediated mechanism of stress adaptation. Supplementation with EVs derived from heat-stressed cells promoted a protective response to subsequent heat stress in recipient GCs. Moreover, the addition of heat-stressed granulosa cells to the in vitro maturation (IVM) medium in bovine oocytes reduces ROS accumulation, enhances mitochondrial function, and alters the expression of stress-associated genes during heat stress, resulting in improved oocyte survival and viability and having a long-lasting positive effect on the resulting blastocysts [7]. Similarly, Saeed-Zidane et al. [8] observed that oxidative-stressed bovine GCs released exosomes enriched with mRNA transcripts of Nrf2 and key antioxidant molecules that modulated the expression of oxidative stress response markers in recipient GCs, indicating that EVs may serve as key mediators of intercellular communication and stress resilience within the follicular microenvironment.

Therefore, the application of an in vitro GCs culture model would enable the study of the effect of oxidative stress on this specific follicular cell type and their response by releasing EVs-coupled molecules into the extracellular space that authentically represent the molecular signatures associated with oxidative injury. Previous studies have demonstrated that H_2_O_2_ exposure reliably induces hallmark features of oxidative stress in GCs, including elevated intracellular ROS, mitochondrial dysfunction, and activation of apoptotic pathways [8]. While this model reliably reproduces the cellular consequences of oxidative damage, the contribution of EV-associated miRNAs to the overall granulosa cell response under oxidative stress has yet to be fully elucidated.

Therefore, we hypothesized that oxidative stress in GCs alters the miRNA cargo within EVs, potentially reflecting and mediating the cellular response to oxidative damage. The aim of this study was to characterize the miRNA profiles of EVs derived from bovine GCs subjected to experimentally induced oxidative stress using H₂O₂. By comparing the EVs miRNA cargo from stressed and non-stressed cells, we sought to identify differentially expressed miRNAs associated with oxidative stress pathways, with the broader goal of uncovering potential regulatory molecules involved in follicular adaptation or dysfunction under adverse conditions.

## Materials and methods

### Chemicals and suppliers

All chemicals and reagents used in this study were purchased from Sigma Chemical Co (St. Louis, MO, USA) except where otherwise indicated.

### Experimental design

**Fig. 1:**
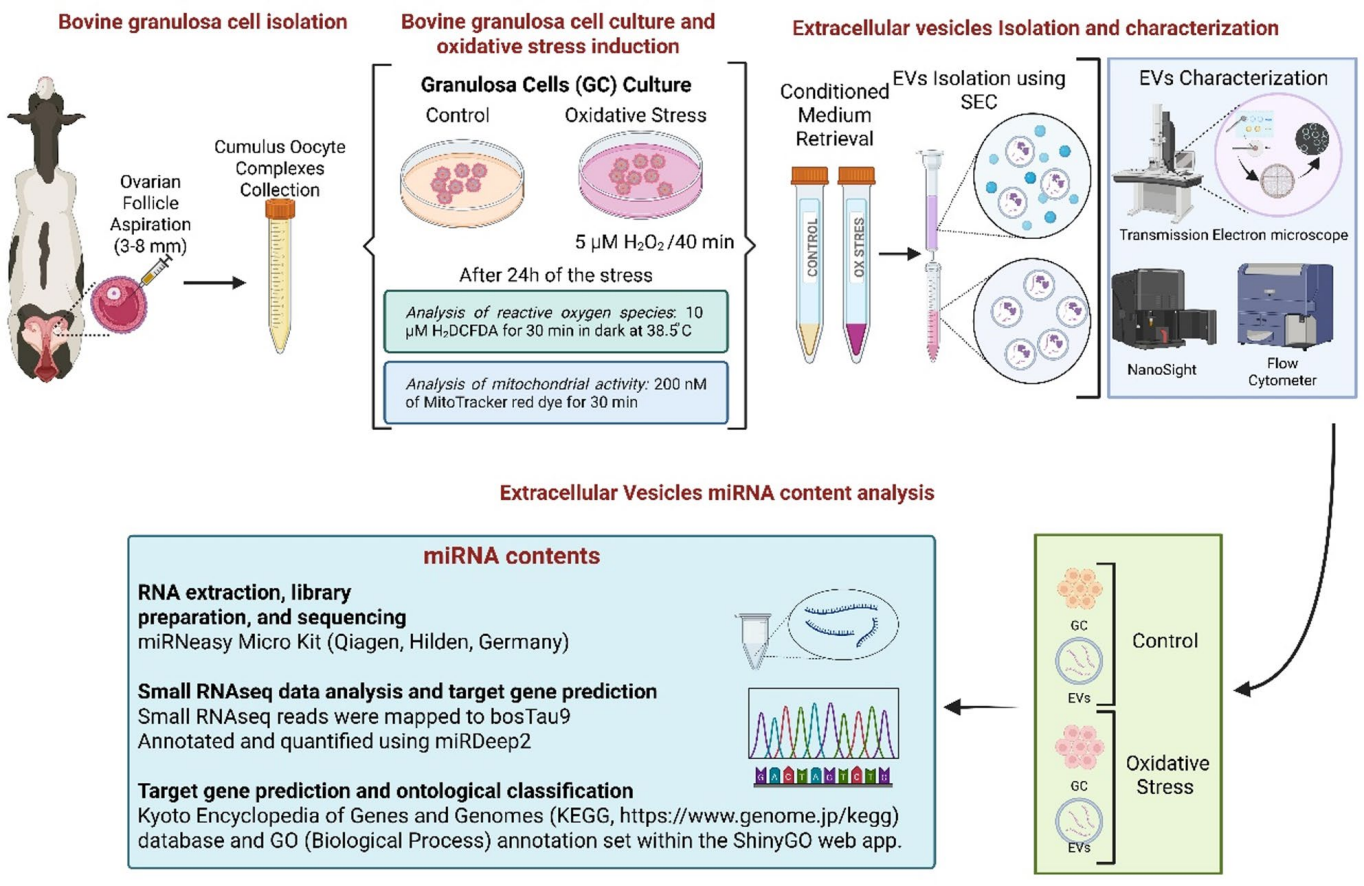
Experimental workflow for oxidative stress induction and extracellular vesicle analysis in bovine granulosa cells. Bovine granulosa cells were cultured in vitro and exposed to oxidative stress or control conditions. After recovery, stress induction was assessed by reactive oxygen species and mitochondrial activity. Extracellular vesicles were isolated from conditioned media by size-exclusion chromatography, characterized, and their miRNA profiles analyzed by small RNAsequencing

Schematic overview of the experimental design shown in Fig. 1.

### Bovine granulosa cell isolation

Bovine ovaries from cyclic crossbred females were collected from a local slaughterhouse (Escorxador Sabadell, S.A., Sabadell, Spain). Approximately 50 ovaries were obtained during the cold months, with each experimental replicate corresponding to a single collection day. Ovaries were transported to the laboratory in saline solution (0.9% NaCl) at 35–37 °C within 1 h. Follicular fluid was aspirated using an 18 g needle from 3 to 5 mm follicles. Aspirated follicular fluid was transferred into a 15 mL tube and cumulus oocyte complexes were let to settle. Then, the supernatant was transferred to a new 15 mL tube with 1:1 phosphate-buffered saline (PBS) and centrifuged at 700 ×g for 7 min. The resulting cell pellet was resuspended in 500 µl RBC lysis buffer for 1 min. After that, 3 mL of PBS was added, and the dilution was centrifuged at 500 *x g* for 7 min. Then, the supernatant was carefully removed, and the cell pellet was resuspended with 500 µL. Resuspended cells were mechanically individualized by passing them 5–10 times through a 25 G needle mounted in 1 mL syringe. Following this, Trypan-blue exclusion approach was used to assess the viability of cells following manufacturer instructions.

### Bovine granulosa cell culture and oxidative stress induction

Granulosa cells were cultured in 35 mm petri dishes. Briefly, 3.75 × 10^5^ live cells per dish were seeded in 3 mL of TCM-199 supplemented with 10% fetal bovine serum (FBS) and antibiotic-antimycotic (100 x). Cells were incubated at 38.5 °C in a humidified incubator with a 5% CO_2_ atmosphere until sub-confluency is attained (∼ 4 days) changing the media every other day. Following this, cells were frozen in FBS supplemented with 10% DMSO at -80 °C and stored for a maximum period of 2 months. The frozen cell stock was generated from four independent biological replicates, each comprising granulosa cells pooled from 45 animals. After thawing, cells were cultured in TCM-199 supplemented with 10% exosome depleted fetal bovine serum (FBS -) (System Biosciences, CA, USA) until subconfluency (∼ 2 days). Then, half of the plates were exposed to 5 µM H_2_O_2_ in PBS for 40 min. Following this, oxidative stressed cells and control cells were cultured for 24 additional hours. After that, the spent culture media were collected for EVs isolation, and the cells were harvested using 0.25% Trypsin–EDTA (ThermoFisher, USA) for further analysis. Granulosa cell RNA-seq, EV enrichment, and EV RNA-seq analyses were performed on three independent replicates derived from the frozen cell stock, and all replicates were processed and analyzed independently.

### Analysis of reactive oxygen species

After 24 h of the stress, sub-confluent cells ROS levels were determined using 2′, 7′-dichlorofluorescin diacetate (H_2_DCFDA) (Invitrogen, USA) according to manufacturer ´s instructions with some modifications. Briefly, GCs from each group were incubated with of 10 µM H_2_DCFDA for 30 min in dark at 38.5 °C. Afterwards, incubated cells were washed twice with PBS and images were captured in an Olympus BX50 with excitation at 460 nm. For each experimental condition, a total of four biological replicates were analyzed. Within each replicate, three independent culture dishes were evaluated, and four randomly selected fields per dish were captured. Regions of interest (ROIs) were defined using an automated threshold-based segmentation approach in ImageJ. For each field, measurements of area and integrated density (IntDen) were obtained, and background fluorescence was measured in cell-free regions. Fluorescence intensity was calculated using the following formula: Relative fluorescence = IntDen − (area of selected ROI × mean fluorescence of background readings). Fluorescence intensities were expressed in arbitrary units (AU).

### Analysis of mitochondrial activity

After 24 h of the stress, cells were incubated with 200 nM of MitoTracker red dye (MitoTracker1 Red CMXRos, M7512; Invitrogen) for 30 min. After washing twice with PBS, cells were fixed with 4% paraformaldehyde overnight at 4 °C. Fixed cells were mounted with VectaShield containing 125 ng/mL DAPI (Vectorlabs, Burlingame, CA, USA). Images were acquired under a laser scanning confocal microscope Leica TCS SP5 (Leica Microsystems CMS GmbH, Mannheim, Germany). Within each replicate, three independent coverslips were evaluated, and four randomly selected fields per coverslip were captured. Regions of interest (ROIs) were defined using an automated threshold-based segmentation approach in ImageJ. For each field, area and integrated density (IntDen) were measured, and background fluorescence was determined from cell-free regions. Fluorescence intensity was calculated as: Relative fluorescence = IntDen − (area of selected ROI × mean fluorescence of background readings). Fluorescence intensities were expressed in AU.

### Extracellular vesicles separation from the spent cell culture media by size exclusion chromatography

EVs were enriched by SEC (Pure EVs^®^, HBM-PEV; Hansa BioMed Life Sciences), according to [10] with minor modifications. Briefly, spent media collected from GCs cultures was centrifuged at 300 ×g for 7 min to remove cellular debris and other large particles. Then, the supernatant was centrifuged at 10.000 ×g for 30 min at 4 °C to further remove debris and large vesicles. After that, the supernatant was filtered with a 0.22 µm filter. For SEC isolation, columns were washed with 30 mL PBS without calcium and magnesium (PBS−) and then sample was loaded onto the top of a SEC column, and when the sample was completely within the column, 11 mL PBS− were loaded. The first 3.0 mL were discarded and the following 2.5 mL corresponding to EVs-enriched fractions were collected. This procedure yields an EVs-enriched fraction that may include co-enriched non-vesicular components [6]. Next, to concentrate the EVs-enriched preparation, 10 K molecular weight cutoff (MWCO) Pierce protein concentrators (ThermoFisher, MA, USA) were used. The sample was centrifuged four times at 2000 ×g for 15 min at 4 °C to achieve a final volume of 100 µL.

### Extracellular vesicles characterization

EV- containing preparation from GCs culture media were characterized using NTA, TEM, and FC following the Minimal Information for Studies of Extracellular Vesicles (MISEV) 2018 guidelines [11]. FC was performed as previously described [12], while NTA and TEM followed the methodology described by Cañon-Beltran et al. [10] with minor modifications. Additionally, we have submitted all relevant data of our experiments to the EV-TRACK knowledgebase (EV-TRACK ID: EV260005) [13]. 

#### Transmission electron microscopy

Five microliters of each EVs preparation were diluted in 45 µL, and then two 25 µL droplets of EVs preparations were allowed to adsorb onto formvar/carbon-coated 200 mesh copper grids (Agar Scientific, Essex, UK) for 1 min at room temperature. Grids were then washed twice with distilled water. For negative staining, grids were transferred to a 50 µL droplet of 2% uranyl acetate for 20s, followed by blotting the excess liquid, and air-dried. TEM visualizations were performed using a JEOL JEM1010 (100 kV) transmission electron microscope (Jeol Ltd., Tokyo, Japan) equipped with a Megaview II CCD camera integrated into iTEM Olympus Soft Imaging Solutions software (Olympus, Tokyo, Japan). As a negative control, PBS- used for elution and carbon-coated grids treated with fixative alone were processed in parallel and imaged under identical conditions.

#### Nanoparticle tracking analysis

 Analysis of concentration and size distribution of EVs was performed using a NanoSight LM-10 system equipped with a CCD video camera and particle-tracking software NTA 3.1 Build 3.1.45 (NanoSight Ltd., Minton Park, UK). Five microliters of EVs preparation were diluted in 95 µL of filtered PBS−. The NTA measurement conditions were detection threshold 2–3, camera level 13, temperature 22 °C and measurement time 60s. Three recordings were performed for each sample.

#### Flow cytometry

The analysis was conducted in accordance with the recommendations of the International Society of Extracellular Vesicles (MIFlowCyt-EV) [11], using the high-sensitivity flow cytometer CytoFLEX S (Beckman Coulter), equipped with violet (405 nm), blue (488 nm), yellow (561 nm), and red (638 nm) lasers. The cytometer was operated in low-flow mode (10 µL/min), with a minimum acquisition of 10,000 events per sample. Filtered distilled water (FDW: 0.1 µm filtered), was used as the sheath fluid. Continuous washing with FDW was performed every 2–3 EVs preparation samples. The optical configuration was optimized to use side scatter (SSC) detection from the 405 nm laser (v-SSC). Both forward scatter (FSC) and v-SSC were set to logarithmic scales, and fluorescence channels were adjusted to logarithmic gains. The EVs detection region was initially delineated using recombinant exosomes tagged with GFP (1 × 10^6^ EVs/mL; SAE0193, Merck) and analyzed in the vSSC/FSC channels setting the threshold at 800 and gating parameters to include over 90% of the total events [12]. For EVs examination, all events within this region were included. Anti-CD63-FITC, anti-CD9-PEVio615 and anti-CD81-PEVio770 antibodies were included for the detection of canonical EV-associated tetraspanins. EVs preparation samples were diluted in 0.1 µm-filtered PBS (FPBS)to a final concentration of 1–2 × 10^6^ EVs/mL and incubated with CellTrace CFSE (0.1 µM, Thermo Fisher) and tetraspanin antibodies (1:50), including anti-CD63-FITC (130-118-076, Miltenyi Biotec; 488 nm excitation, B1 525/25 nm detection), anti-CD9-PEVio615 (130-118-811, Miltenyi Biotec; 561 nm excitation, R2 615/20 nm detection) and anti-CD81-PEVio770 (130-107-922, Miltenyi Biotec; 405 nm excitation, B3 450/50 nm detection). FPBS was employed to prepare working solutions. The incubation was performed for 30 min at 37 °C, after which 200 µL of FPBS was added to stop the reaction. Prior to incubation, working solutions of CFSE and antibodies were centrifuged three times (17,000 × g, 10 min each) to pellet any residual particles. Fluorescence controls were carried out to verify the specificity of the CFSE and CD9, CD63, and CD81 antibodies, using separate buffers for each. Implemented controls included FPBS, unstained EVs preparation, and antibody mixes in the absence of EVS, as well as only CFSE. Data acquisition and analysis were performed using the CytoFLEX S cytometer and CytoExpert software.

Finally, the presence of residual cellular contamination was evaluated by flow cytometry both before and after gentle sample permeabilization, using antibodies against BSA and calnexin. Permeabilization was carried out using a mild detergent treatment (0.1% Triton X-100 in PBS for 30 min at room temperature). After this step, the samples were incubated for 1 h at room temperature with primary antibodies diluted in the same buffer (anti-BSA mouse monoclonal antibody, 1:1000, Sigma-Aldrich SAB1200688; anti-calnexin rabbit polyclonal antibody, 1:1000, Abcam AB22595), followed by a 30 min incubation with the corresponding secondary antibodies, anti-mouse Alexa Fluor 488 and anti-rabbit Alexa Fluor 647 (Abcam). Specificity was confirmed through multiple negative controls, including: (i) permeabilization buffer alone; (ii) secondary antibodies without primaries; (iii) permeabilized EVs without antibodies; and (iv) unfiltered reproductive fluid, known to contain cellular debris, processed in parallel. Data were analyzed using the EV-optimized configuration incorporating FSC/SSC-violet gating.

### Extracellular vesicles miRNA content analysis

#### RNA extraction, library construction, quality control and sequencing

Total RNA from GCs was isolated using the mirVana™ miRNA Isolation Kit (Thermo Fisher Scientific, Waltham, MA, USA) following the protocol for cells in suspension. The miRNeasy Micro Kit (Qiagen, Hilden, Germany) was used to isolate total RNA from EVs, following the manufacturer’s instructions. Library-prep, sequencing and trimming of the raw samples were done by the sequencing provider (Novogene Corporation Inc.). The RNA concentration and size distribution were evaluated using an Agilent RNA 6000 Pico kit on an Agilent 2100 Bioanalyzer (Agilent Technologies, Santa Clara, CA, USA). Next-generation sequencing (NGS) small-RNA libraries were created with a QIAseq miRNA Library Kit (Qiagen), following the manufacturer’s instructions. During library preparation, 3′ and 5′ adaptors were ligated to the respective ends of small RNA, followed by reverse transcription and PCR amplification to generate double-stranded cDNA libraries with insertions between 18 ~ 40 bp that were purified and selected. Quality and quantity assessments of the libraries were done using the Agilent DNA High Sensitivity kit (Agilent Technologies) and a Qubit DNA HS Assay Kit (Thermo Fisher Scientific), respectively. Subsequently, the libraries were combined in equimolar ratios and then sequenced as single-end reads on a NovaSeq6000 sequencing instrument (Illumina, Inc., San Diego, CA, USA), according to effective library concentration and data amount (20 M reads per sample) using a single-end 50 bp (SE50) sequencing strategy. Raw read files were evaluated, filtered and trimmed by the sequencing provider (Novogene Corporation Inc). The number of total and quality-controlled reads for each individual library in both GC and EVs is summarized in Additional file 1.

Quality of the clean (processed) small RNAseq reads was confirmed using FASTQC (Babraham Bioinformatics) and SeqKit prior to the downstream analyses. Read length statistics are provided in Additional file 2.

#### Small RNAseq data analysis and target gene prediction

The processed small RNAseq reads were mapped to the cattle reference genome (bosTau9), annotated and quantified using the program miRDeep2 [14] based on bovine precursor and mature miRNAs available from the mirBase database (release 22 [15]). The percentage of reads in the samples that map to other small RNAs was determined by mapping against bovine non-coding sequence libraries (excluding miRNAs) (ARS-UCD2.0) using bowtie and reported in Additional file 3. Normalization of miRNA read counts and differential expression (DE) analysis between control and oxidative samples was done using DEseq2 [16]. Low counts were removed using the DESeq2 independent filtering step. Differential expressions were tested using the DESeq2 Wald-test, comparing the different groups. DESeq2 uses raw counts as input and normalizes them for differential expression analysis using DESeq2 median of ratios [17] and adjusts the p-value for multiple testing by using the Benjamini-Hochberg method (False discovery rate (FDR)). Log2 fold change (LFC) adaptive shrinkage was performed in DESeq2 using the ashr package (v2.2). Cutoffs for DE was set at FDR < 0.05, and LFC > 1. Sequencing data are available on NCBI’s Gene Expression Omnibus (GEO) with the accession number GSE304292. Target genes for significantly differentially abundant miRNAs were obtained from TargetScan (v8.0) [18, 19] and miRWalk databases [20]. Of the target genes found we produced a list of high confidence target genes based on the TargetScan cumulative weighted context score (or “CWCS”) of − 0.4 or lower. Pathways and biological processes (BP) of target genes were determined from the Kyoto Encyclopedia of Genes and Genomes (KEGG, https://www.genome.jp/kegg) database and GO (Biological Process) annotation set within the ShinyGO web app [21].

### Statistical analysis

To perform all statistical tests and graphs, the software GraphPad Prism 9 (GraphPad Software, CA, USA) for Mac and R software V 4.1.1 (R Core Team, Vienna, Austria) were used. Data were first checked for normality using the Shapiro-Wilk’s test and for homogeneity of variances using the Levene test. After checking data normality and homoscedasticity, statistical differences in concentration and size of the EVs and in ROS levels and mitochondrial activity between treatment groups were analyzed by an unpaired t-test. The mean ± standard deviation (SD) and mean ± standard error of the mean (SEM) is used to express data, respectively. Significance was set at *P* ≤ 0.05. miRNAseq results were statistically analyzed using R/Bioconductor packages.

## Results

### Effect of oxidative stress on ROS accumulation and mitochondrial activity

To assess the effect of oxidative stress on ROS accumulation in GC, fluorescence intensity was measured in control and oxidative stressed GC. As shown in Fig. 2.1, GC exposed to oxidative stress exhibited a significant increase in ROS levels compared to the control group at 24 h post-treatment. Mitochondrial activity was evaluated under control and oxidative stress conditions using fluorescence-based detection. As depicted in Fig. 2.2, oxidative stress significantly decreased mitochondrial activity compared to the control group at 24 h post-treatment.

**Fig. 2:**
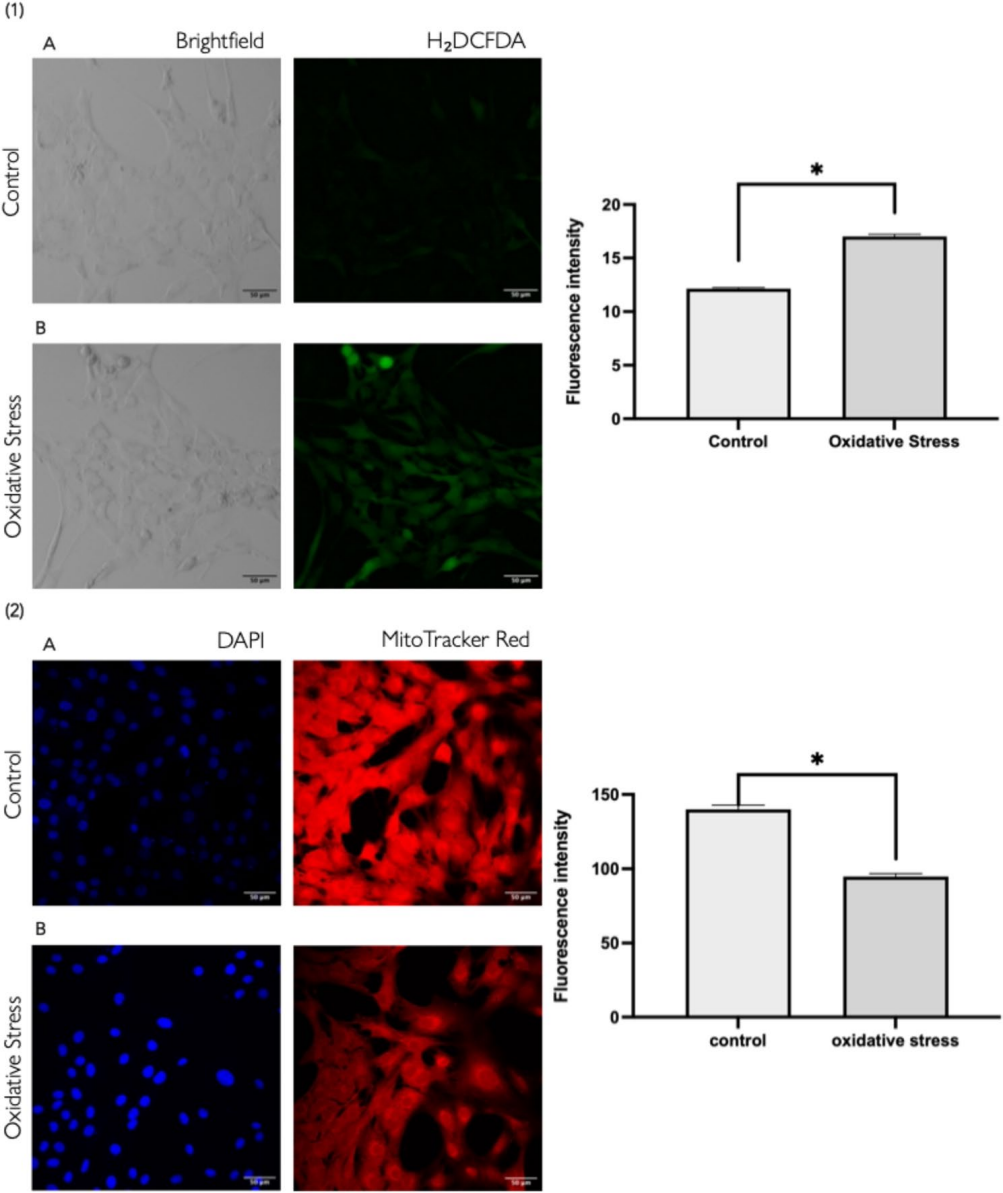
Impact of H_2_O_2_ treatment in bovine granulosa cells. **1** Quantification of ROS fluorescence intensity and representative fluorescence microscopy images of intracellular ROS levels in control (A) and OS (B) granulosa cells, detected using 2′,7′-dichlorofluorescin diacetate (H_2_DCFDA). **2** Quantification of mitochondrial activity and representative fluorescence microscopy images of control (A) and OS (B) granulosa cells, assessed using MitoTracker Red CMXRos. Data are presented as mean ± SEM from three independent biological replicates. * Indicates statistically significant differences (*P* < 0.05) among groups. Scale bar: 50 μm

### Characterization of granulosa cell derived EVs

**Fig. 3:**
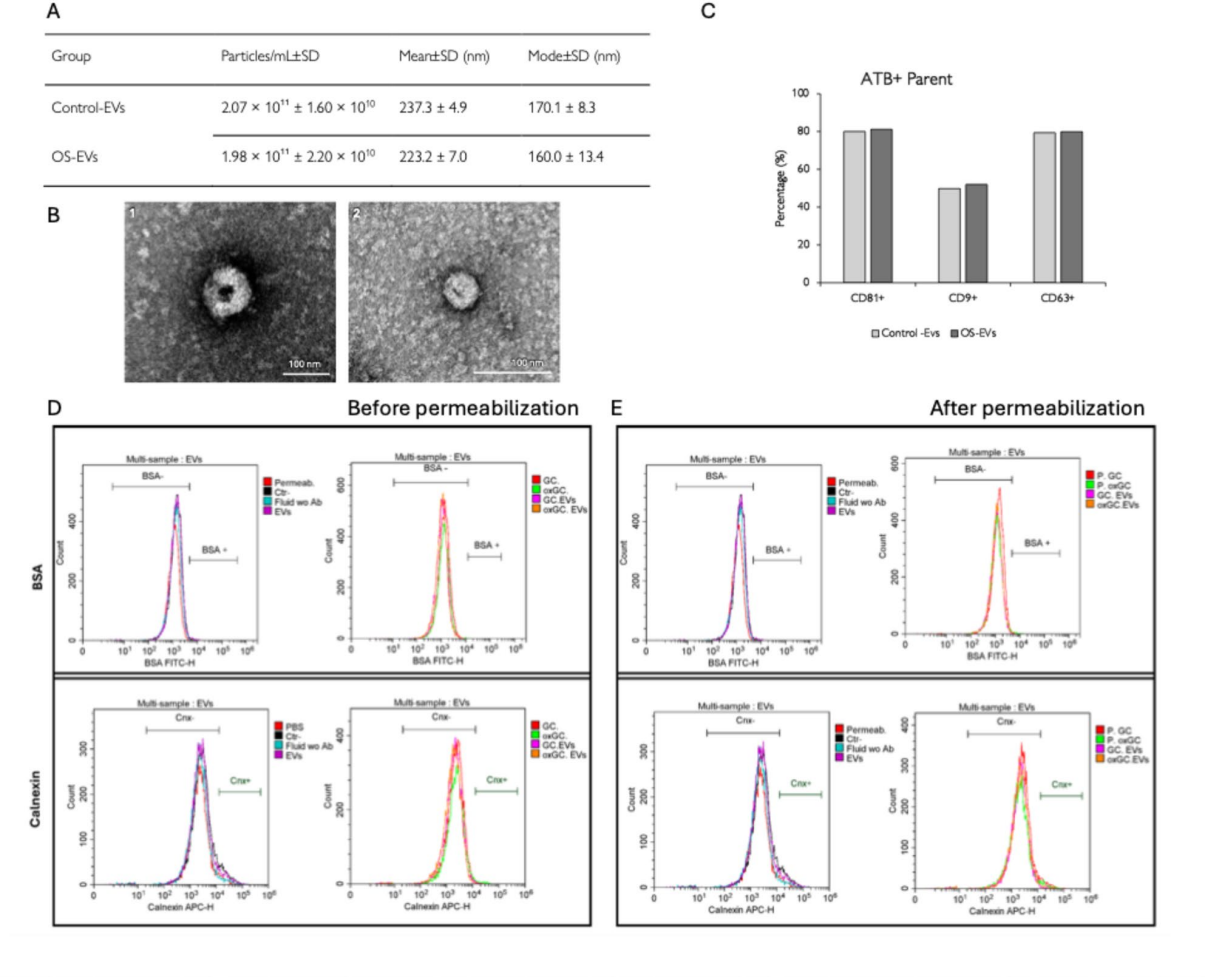
Characterization and evaluation of cellular contamination in EV-enriched samples derived from control and oxidative-stressed granulosa cells. **A** Concentration, mean and modal size of GC-EVs from control (Control-EVs) and OS (OS-EVs) cells. **B** Representative transmission electron microscopy (TEM) images from EVs. The images show [1] EVs isolated from control (Control-EVs) and OS (OS-EVs) granulosa cells. Scale bar = 100 nm. **C** Flow cytometry confirmed the presence of the canonical EV tetraspanins CD81, CD9, and CD63 in both experimental conditions. **D** Flow cytometry analysis of BSA and calnexin in non-permeabilized conditioned media derived from control and oxidative-stressed granulosa cell cultures and their corresponding EV preparations. **E** Flow cytometry analysis of BSA and calnexin in permeabilized conditioned media derived from control and oxidative-stressed granulosa cell cultures and their corresponding EV preparations. Control granulosa cells (GC, red); Oxidative-stressed granulosa cells (oxGC, green); CT-EVs (GC.EVs, pink); OS-EVs (oxGC.EVs, orange); permeabilization buffer alone (Permab, red); secondary antibodies diluted in permeabilization buffer without primaries (Ctr–, black); conditioned media incubated only with secondary antibodies (Fluid wo 1Ab, blue)

Vesicle size and concentration were assessed by NTA. Control EVs exhibited a concentration of 2.07 × 10^11^ ± 1.60 × 10^10^ particles/mL, with a mean diameter of 237.3 ± 4.9 nm and a mode of 170.1 ± 8.3 nm. Similarly, OS EVs showed a concentration of 1.98 × 10^11^ ± 2.20 × 10^10^ particles/mL, with a mean diameter of 223.2 ± 7.0 nm and a mode of 160.0 ± 13.4 nm. No differences in particle size or concentration were observed between Control-EVs and OS-EVs (Fig. 3A). The size and morphology of EVs were further characterized by TEM, revealing their characteristic cup-shape (Fig. 3B). Control micrographs of PBS and fixative-treated grids showed no vesicular structures, confirming the specificity of the observed EVs (Additional Fig. 1).

Flow cytometry analysis demonstrated the presence of membrane-intact EVs, as indicated by CFSE positivity, with 87.70% of Control-EVs and 83.64% of OS-EVs being CFSE-positive. In addition, immunofluorescence-based flow cytometry detected the EV-associated tetraspanins CD81, CD9, and CD63 in both groups (Fig. 3C). In Control-EVs, the percentage of EVs positive for CD81, CD9, and CD63 was 80.06%, 49.74%, and 79.44%, respectively, with corresponding fluorescence intensities of 2066.6 AU (CD81), 1112.0 AU (CD9), and 3034.1 AU (CD63). Comparable values were observed in OS-EVs, with 81.24% CD81-positive, 52.00% CD9-positive, and 79.96% CD63-positive EVs, and fluorescence intensities of 1852.0 AU (CD81), 1114.9 AU (CD9), and 3094.3 AU (CD63). These findings further validate the effectiveness of the isolation protocol. Potential residual cellular contamination in EVs-enriched fractions was evaluated by flow cytometry using antibodies against BSA and calnexin. No detectable fluorescence signal was observed under any condition, indicating the absence of cellular contaminants in EVs preparations derived from either control or oxidatively stressed granulosa cells, including after membrane permeabilization (Fig. 3D and E).

### MicroRNA expression in granulosa cells following OS exposure

Small-RNA-seq analysis revealed an average of 23 million and 24 million raw reads per library for the Control and the OS groups, respectively. On average, 7.8% of reads specifically annotated as bovine miRNAs according to the mirBase database (Additional file 4). A total of 716 were expressed in the Control group, while 775 miRNAs were detected in the OS group.

**Fig. 4:**
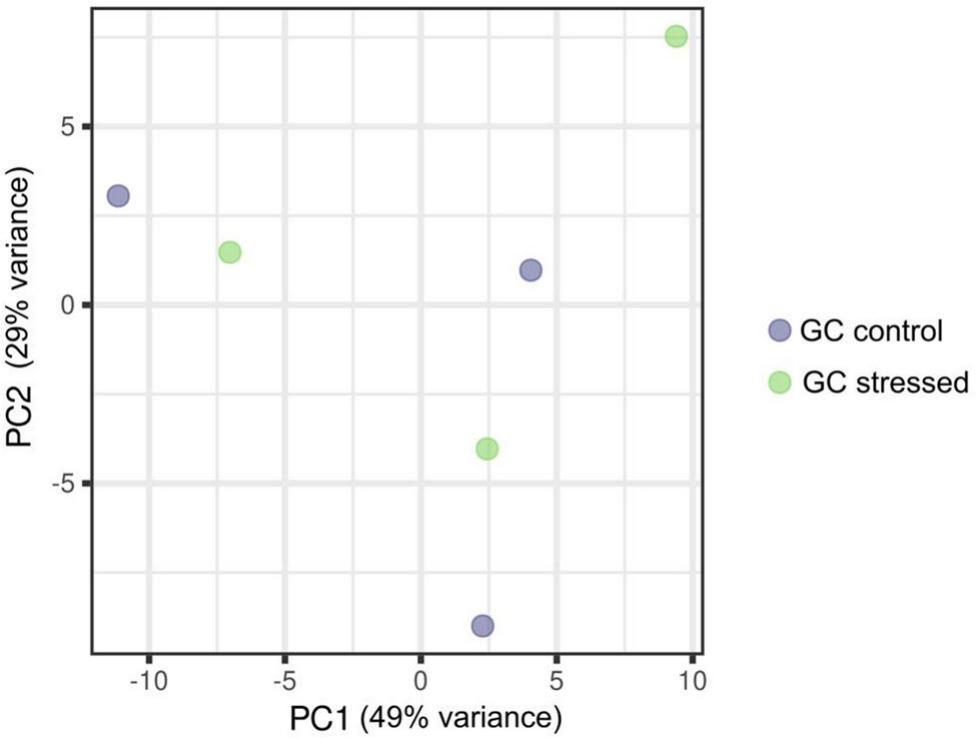
Principal component analysis (PCA) plot of GC samples from the control and OS groups. Control (blue dots) and OS (green dots) samples

Principal component analysis (PCA) shows no clear clustering according to treatment group, indicating no global miRNA expression differences (Fig. 4). A detailed summary of all detected miRNAs in both Control and OS groups is presented in Additional file 5. No differential expression was found between treatments in GCs miRNAs. 

### Top expressed miRNAs in granulosa cells

The top five most abundant miRNAs were: let-7i, miR-21-5p, miR-148a, miR-151-3p, and miR-27b in both the Control and OS groups (Table 2).

**Table 1:** Top abundant miRNAs in granulosa cells

Name	Control group normalized counts	Oxidative stress group normalized counts
bta-let-7i	211,095.31	226,112.25
bta-miR-21-5p	197,600.60	207,859.01
bta-miR-148a	163,319.09	201,847.79
bta-miR-151-3p	212,913.40	192,554.50
bta-miR-27b	86,411.82	82,741.09

#### Gene ontological classification in granulosa cells and pathway overrepresentation analysis of the target genes of top abundant GC miRNAs

Pathway enrichment and GO terms analysis was performed for gene targets of the top five most abundant miRNAs in both OS and Control samples using ShinyGO (https://bioinformatics.sdstate.edu/go/).

Table 3 presents the top 10 KEGG pathways enriched for these target genes, which include key signaling pathways such as MAPK, TGF-β, FoxO, Ras, EGFR and PI3K-Akt (Additional file 6). Additionally, Additional file 7 provides a comprehensive overview of GO - BP potentially regulated by these miRNAs. Notable processes include protein phosphorylation, RNA transcription, cell differentiation, regulation of gene expression and cell cycle (Table 4).

**Table 2:** Functional classification of target genes of top 5 miRNAs in GCs. Top 10 enriched KEGG pathways

Pathway	Fold enrichment	FDR
MAPK signaling pathway	2.7	1.10E-06
TGF-beta signaling pathway	3.9	4.70E-06
FoxO signaling pathway	3.3	2.60E-05
Signaling pathways regulating pluripotency of stem cells	3.2	3.00E-05
Ras signaling pathway	2.4	4.10E-04
EGFR tyrosine kinase inhibitor resistance	3.4	1.90E-03
PI3K-Akt signaling pathway	1.9	2.70E-03
Insulin signaling pathway	2.5	5.50E-03
TNF signaling pathway	2.6	7.70E-03
Regulation of actin cytoskeleton	2.1	7.90E-03

FDR, false discovery rate

**Table 3:** Functional classification of target genes of top 5 miRNAs in GCs. Top 10 enriched GO-terms (Biological process)

Pathway	Fold enrichment	FDR
Positive regulation of transcription by RNA polymerase II	2.3	2.40E-19
Protein phosphorylation	2.6	2.90E-06
Chromatin remodeling	2.5	6.50E-05
Regulation of transcription by RNA polymerase II	1.6	6.70E-05
Phosphorylation	2	1.00E-04
Signal transduction	1.7	1.50E-04
Positive regulation of cell population proliferation	2.1	1.50E-04
Positive regulation of gene expression	2.1	3.50E-04
Cell differentiation	1.7	1.60E-02
Positive regulation of protein phosphorylation	2.4	1.60E-02

FDR, false discovery rate

### Differential miRNA expression in extracellular vesicles derived from granulosa cells exposed to oxidative stress 

Small-RNA-seq analysis yielded an average number of 18 million and 23 million raw reads per library for EVs samples from the Control and OS groups, respectively. On average, 15.79.% of reads were specifically annotated as bovine miRNAs according to the mirBase database (Additional file 4).

**Fig. 5:**
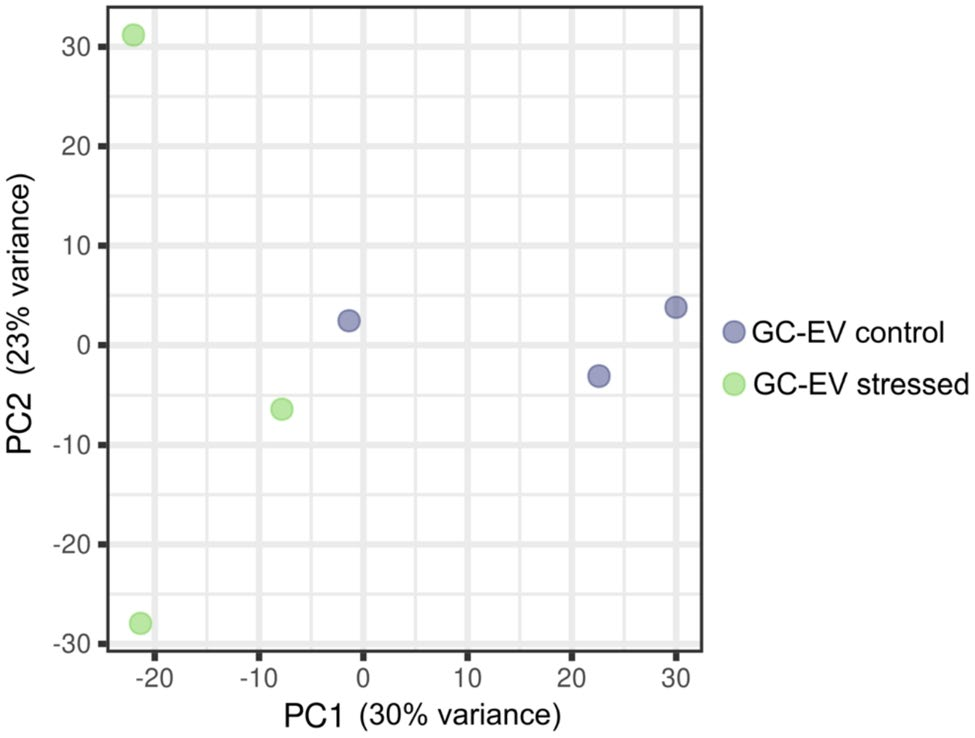
Principal component analysis (PCA) plot of GC-EV samples from the control and OS groups. Control (blue dots) and OS (green dots) samples

A total of 351 miRNAs were detected in the Control-EVs group, and 514 miRNAs in the OS-EVs group. PCA revealed a clear grouping of biological replicates, indicating a generalized effect of the treatment on miRNA abundance (Fig. 5; Additional file 8).

**Fig. 6:**
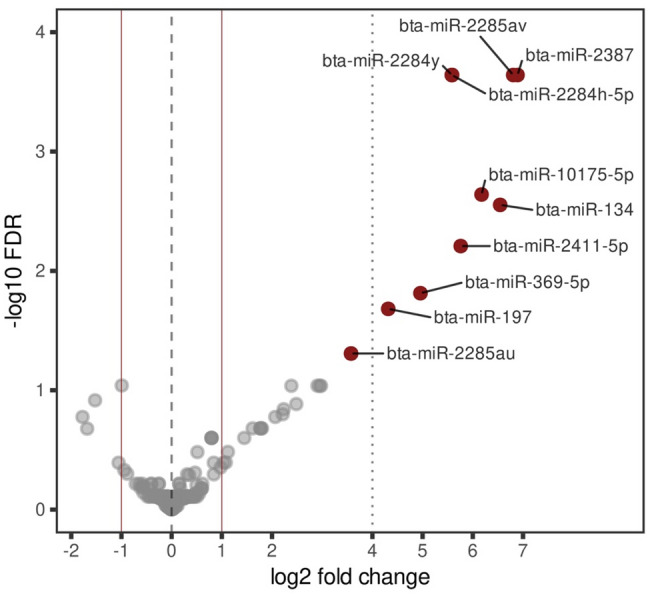
Visualization of differential expression analysis of miRNAs in EVs. Volcano plot of differential expression data of miRNAs in EVs from control versus OS groups. Significantly higher abundant mature miRNAs are highlighted in red and labelled

Differential miRNA expression results between Control-EVs and OS-EVs were visualized using a Volcano plot (Fig. 6).

Differential expression analysis identifies 10 miRNAs: including miR-2387, miR-2285av, miR-134, miR-10175-5p, miR-2411-5p; miR-2284y, miR-2284 h-5p, miR-369-5p, miR-197 and miR-2285au as significantly more abundant in the OS-EVs group compared to the Control-EVs group (LFC) ≥ 1, FDR < 0.05) (Table 5). Target genes for these 10 differentially expressed miRNAs were predicted using TargetScan and miRWalk databases, with a summary of the number and IDs of target genes provided in Additional file 9.

**Table 4:** Differentially expressed miRNAs in extracellular vesicles derived from granulosa cells under oxidative stress versus control conditions

Name	Sequence	LFC	FDR
bta-miR-2387	UGGAAGGCCUGGCUUUGCAGCG	6.89	0.00
bta-miR-2285av	AAAAACUGAAUGAAAUUCUUGG	6.80	0.00
bta-miR-134	UGUGACUGGUUGACCAGAGUGG	6.54	0.00
bta-miR-10175-5p	UGGAGAGAACAGGUGGCUUU	6.17	0.00
bta-miR-2411-5p	UGGAGUGACUGUCAGAUGCAGCCA	5.76	0.01
bta-miR-2284y	AAAAGUUGGUUCGGGUUUUU	5.59	0.00
bta-miR-2284 h-5p	GAAACAUUCACUCGGGUUUUU	5.58	0.00
bta-miR-369-5p	AUCGACCGUGUUAUAUUCGC	4.96	0.02
bta-miR-197	UUCACCACCUUCUCCACCCAGC	4.31	0.02
bta-miR-2285au	AAAAACUGAAUGAAAUUCUUGG	3.57	0.05

LFC, fold change; FDR, false discovery rate

### Gene ontological classification of miRNA target genes in extracellular vesicles derived from granulosa cells exposed to oxidative stress and pathway overrepresentation analysis

GO enrichment analyses were performed on high confidence TargetScan gene targets of the differentially expressed miRNAs from the Control-EVs and OS-EVs groups. These target genes are associated with key biological processes such as angiogenesis, cell migration, anatomical structure morphogenesis and L-glutamate import across plasma membrane (Table 6). Furthermore, we used the mirWALK intrinsic gene target pathway overrepresentation analysis tool to test for KEGG pathway overrepresentation among the mirWALK target genes of the differentially expressed miRNAs (Table 7; Additional file 9). Key signaling pathways, such as WNT, MAPK and Oxytocin signaling are among the KEGG terms that are overrepresented.

**Table 5:** Top 10 enriched GO-terms (Biological process) in high confidence TargetScan target genes of differentially expressed miRNAs in EVs derived from granulosa cells exposed to oxidative stress compared to control conditions

Term	Fold enrichment	FDR
Angiogenesis	2.72	0.02
Positive regulation of L-glutamate import across plasma membrane	50.24	0.02
Anatomical structure morphogenesis	1.68	0.02
Regulation of vasoconstriction	6.53	0.02
Blood vessel morphogenesis	2.52	0.02
Anatomical structure formation involved in morphogenesis	2.09	0.02
Circulatory system development	2.08	0.02
Vasculature development	2.28	0.05
Cell migration	1.84	0.05
Blood vessel development	2.29	0.05
Regulation of L-glutamate import across plasma membrane	25.12	0.05
Choline transport	25.12	0.05
Vasoconstriction	5.15	0.05
Cell motility	1.75	0.05
Localization of cell	1.75	0.05

FDR, false discovery rate

**Table 6:** Top 10 enriched KEGG pathways in mirWALK enrichment analysis of significantly higher abundant miRNAs in EVs derived from granulosa cells exposed to oxidative stress compared to control conditions

Pathway	*n* genes	*n* pathway	FDR
Pathways in cancer	201	543	< 0.001
MAPK signaling pathway	117	299	< 0.001
Ras signaling pathway	114	246	< 0.001
Endocytosis	103	249	< 0.001
Regulation of actin cytoskeleton	94	228	< 0.001
Axon guidance	84	179	< 0.001
Wnt signaling pathway	76	175	< 0.001
Hepatocellular carcinoma	74	174	< 0.001
Oxytocin signaling pathway	71	153	< 0.001
Phospholipase D signaling pathway	68	153	< 0.001

FDR, false discovery rate

## Discussion

Conditions of negative energy balance, heat stress, or other metabolic challenges are known to increase ROS levels in GCs, thereby exposes the follicular environment to OS. An excess of ROS level causes abnormal GC growth, increased apoptosis, and altered steroidogenic function, which are crucial to the development of cystic ovarian follicles, anestrus, or other reproductive disorders. Previous studies have established H_2_O_2_ as a reliable and reproducible inducer of OS in cultured cells, including granulosa cells. Although hydroxyl radicals (–OH) are more reactive, H_2_O_2_ has a longer half-life, allowing it to diffuse across membranes and interact with various cellular components, including DNA [22]. Its relative stability enables controlled induction of oxidative stress without excessive cytotoxicity, which makes it particularly suitable for in vitro studies. Saeed-Zidane et al. [8] demonstrated that exposure of bovine GCs to 5 µM H_2_O_2_ for 40 min effectively induced oxidative stress, characterized by elevated intracellular ROS levels, and mitochondrial dysfunction, thus providing a validated and moderate stress model. In our study, a similar treatment produced consistent results, with increased intracellular ROS accumulation and reduced mitochondrial activity 24 h post-treatment, confirming that H_2_O_2_ effectively mimics oxidative injury in bovine GCs.

Oxidative or heat stress not only disrupts the intracellular environment of GCs but also changes the secretion and molecular cargo of EVs. GCs under stress release exosomes enriched with antioxidant-related mRNAs, which can influence the stress response in neighboring cells [7, 8]. The characterization of EVs released from bovine GCs under oxidative stress conditions revealed that both Control and OS-treated GCs released EVs with comparable concentration ranges, mean diameters and modal sizes. Previous studies reported a significantly higher number of EVs released under stress, while maintaining a similar [9], or lower size [8] distribution. In this study, while OS did not alter EVs concentrations, a trend toward smaller vesicle sizes was detected. This shift, even if not statistically significant, may reflect adaptive changes in EVs biogenesis pathways. These size changes are thought to be linked to alterations in the molecular machinery involved in EVs biogenesis, potentially influencing the cargo and functional properties of the released vesicle [23].

MicroRNAs have recently been recognized as key post-transcriptional regulators involved in numerous reproductive pathophysiological disorders. MicroRNAs act as post-transcriptional gene-expression regulators by inhibiting protein translation or promoting mRNAs for degradation. Recent studies have demonstrated the role of miRNAs in a wide range of physiopathological processes occurring in the female reproductive system (reviewed in [24]). In particular, several miRNAs have been involved in the regulation of granulosa cell proliferation and differentiation, steroid synthesis and apoptosis [25]. miRNAs have been identified as major regulators under conditions of oxidative stress [26]. Furthermore, accumulating evidence indicates that oxidative stress conditions significantly modulate miRNA expression profiles in GCs across mammalian species [27, 28]. In bovine, Sohel et al. [27] demonstrated that exposure of GCs to H_2_O_2_ induces widespread changes in apoptosis-related miRNAs, with 69 miRNAs found to be differentially expressed using a targeted PCR array approach. In contrast, our study, employing small RNA sequencing on GCs, identified a different subset of oxidative stress-responsive miRNAs. The divergence between the miRNA profiles reported in both studies likely reflects a combination of biological and methodological variables, including differences in H₂O₂ concentration and exposure duration, and miRNA detection platforms (PCR array vs. RNA-seq). Furthermore, accumulating evidence has shown that stress conditions significantly modulates miRNA expression profiles in GC, including those in bovine and porcine species. While in our study GCs were exposed to a mild, acute oxidative stress (5 µM H_2_O_2_ for 40 min), a condition calibrated to induce a transient cellular stress response without triggering widespread cytotoxicity, Sohel et al. [27] applied a considerably higher concentration of H₂O₂ (150 µM for 4 h), consistent with a prolonged and more severe oxidative challenge. These divergent conditions are likely to activate distinct transcriptional and post-transcriptional programs: lower doses may elicit adaptive responses aimed at restoring redox balance and preserving follicular function, while higher doses may predominantly engage apoptotic and cell death pathways [28].

The results of this study demonstrate that this mild oxidative stress resulted in subtle but consistent cellular alterations, which were sufficient to induce changes in EV cargo composition without causing major phenotypic divergence at the cellular level. Although the type of stressor differs, our findings parallel those of Gebremedhn et al. [9], who reported heat stress-induced modifications in EVs-associated miRNAs. Taken together, these studies underscore the responsiveness of EVs miRNA profiles to different forms of cellular stress -heat or oxidative- and suggest a regulatory role for EVs-mediated signaling in granulosa cell adaptation and follicular function under adverse conditions. The ability of GC to dynamically modify their EVs cargo suggests a mechanism by which these cells may coordinate survival strategies within the follicular microenvironment. Notably, the nature of the stressor—oxidative versus thermal—appears to drive the selective enrichment of stress-responsive miRNAs. However, the specific miRNAs and their predicted targets differ between studies, reflecting potentially different molecular adaptation strategies related to the type of stress encountered. In the current study, oxidative stress did not induce any upregulation of miRNAs in GC. However, ten miRNAs—including miR-134, miR-2285av, miR-2284 and miR-197—were significantly upregulated in EVs derived from oxidatively stressed GCs, and their predicted targets were associated with processes such as angiogenesis, cell migration, anatomical structure morphogenesis and L-glutamate import across plasma membrane and pathways such as WNT, MAPK and Oxytocin signaling.

By contrast, Gebremedhn et al. [9] reported 14 differentially expressed miRNAs in heat-stressed GC, with six miRNAs—including bta-miR-2449 and bta-miR-6523a—upregulated, and eight others downregulated. Their analysis of EVs from heat-stressed cells revealed selective enrichment of five miRNAs, including bta-miR-1246 and bta-miR-374a, with target genes clustered in metabolic pathways, circadian rhythm, AMPK signaling, and lipid homeostasis. These findings collectively underscore the stress-specific nature of miRNA packaging into EVs, suggesting that GC deploy distinct regulatory networks in response to different types of cellular insult. While there is convergence at the level of general stress-response pathways, the divergence in miRNA identities and their functional annotations emphasizes the complexity and specificity of the follicular stress response landscape.

Several miRNAs were found to be highly abundant in GCs regardless of oxidative stress status. Among them, miR- let-7i, has been found to have a potential role in regulating follicular function. In porcine, hsa-let-7i has been reported to be upregulated in healthy follicles compared to atretic ones, suggesting an association with follicular viability [29, 30]. In cattle, let-7i is carried by low-density EVs in the follicular fluid, where it targets FASLG to inhibit granulosa cell apoptosis [31]. Additionally, let-7i has been shown to suppress granulosa-luteal cell proliferation and estradiol biosynthesis by directly targeting IMP2 [32]. Let-7i has also been reported to be upregulated by dioscin in models of LPS-induced inflammatory kidney injury where it exerts protective effects by targeting the TLR4/MyD88 signaling pathway and attenuating inflammation, oxidative stress, and apoptosis [33]. These findings suggest that let-7i plays a key role in cellular responses contributing to follicular function by modulating apoptosis, inflammation, and steroidogenesis. MiR-21-5p has been reported to play a complex role in granulosa cell function and follicular dynamics. In mice, a decrease in miR-21-5p promotes IL-1β secretion from theca cells, which in turn inhibits the cAMP–PKA pathway and activates NF-κB signaling in GC. This cascade leads to increased ROS levels, calcium imbalance, mitochondrial damage, apoptosis, and reduced estradiol production [34]. Moreover, studies in pigs have shown that miR-21-5p is the most abundant miRNA in exosomes from follicular fluid, where it enhances glucose uptake by targeting BTG2 and activating the IRS1/AKT pathway [35]. In chicken, miR-21-5p has an increasing concentration in GC with follicle maturation [36]. This miRNA has been associated with both pro-apoptotic and anti-apoptotic processes. While Cao et al. [34] associated it with increased apoptosis in GC, Zhang et al. [37] reported the opposite effect, promoting granulosa cell proliferation and inhibiting apoptosis by downregulating Smad7. Additionally, elevated levels of miR-21-5p levels have been reported in cumulus cells from women classified as poor ovarian responders during IVF procedures [38], suggesting its potential involvement in impaired ovarian function. Notably, its overexpression has also been detected in ovarian cancer [39], further implicating this miRNA in ovarian pathophysiology. MiR-148a contributes to granulosa cell differentiation in chickens [36]. In pigs, miR-148a-3p is the most abundant miRNA in granulosa cell-derived exosomes and has been shown to enhance oocyte developmental competence [40]. In addition to its role in the ovary, miR-148a modulates oxidative stress and inflammation in a sepsis-induced acute kidney injury model, by modulating the CIRC-Ttc3/Rcan2 axis, thereby providing protective effects against cellular damage [41]. In bovine, miR-151-3p has been identified as a key component of uterine fluid EVs [42]. Its high abundance in follicular fluid has been associated with enhanced oocyte maturation and embryonic development, and its supplementation during IVM or culture (IVC) improved blastocyst formation rates [43]. In human mesangial cells, miR-151-3p has been shown to regulate oxidative stress by mediating the protective effects of circNUP98 knockdown under high glucose conditions, through targeting HMGA2, in a model of diabetic nephropathy [44]. miR-27b, an oxidative stress-responsive miRNA, has been detected in granulosa and cumulus cells in the mare with higher expression in mid-estrous follicles compared to pre-ovulatory follicles and it is predicted to target ACVR1 and ID2 known for their importance during follicular development [45]. Additionally, in rats, miR-27b exacerbates intracerebral hemorrhage-induced brain injury by downregulating the Nrf2/ARE pathway, with its inhibition reducing oxidative damage, neuroinflammation, and neuronal death [46]. Altogether, these observations highlight the role of miRNAs in modulating granulosa cell function, oocyte competence, and key pathways involved in apoptosis, inflammation, and steroidogenesis.

Our results demonstrate that oxidative stress significantly alters the miRNA cargo of EVs derived from bovine GC. The higher abundance of miR-134 observed in EVs derived from OS-exposed GC may reflect a broader cellular mechanism of stress adaptation and damage control. miR-134 has been reported to exert tumor suppressive functions by targeting genes such as *EGFR*, *KRAS*, *CDK4* or *STAT5B*, either by repressing their expression directly or promoting apoptosis through activation of caspase-3/7 or repression of Bcl-2 (reviewed in [47]). However, anti-apoptosis effects by targeting WWOX gene and suppressing ERK1/2 pathway activation have also been described [48]. In the present study, miR-134 enrichment in EVs released under oxidative stress conditions may contribute to the modulation of apoptosis and cellular stress responses potentially supporting follicular integrity or adaptive survival mechanisms through paracrine signaling. However, due to the dual nature of miR-134, further studies are warranted to elucidate its specific function in the ovarian follicular microenvironment under oxidative stress. Another abundant miRNA identified in this study is miR-2284, a ruminant specific miRNA [49] associated with cellular response to heat stress [50]. Previous studies have reported divergent expression patterns under heat stress conditions as it was found to be downregulated and linked to heat-induced immune responses in Holstein cows [51], and increased abundance in Tharparkar and Karan Fries cattle [52]. These findings suggest that its expression may be modulated by breed-specific differences in thermotolerance and stress adaptation mechanisms.

miR-197 was also found to be higher abundant in our dataset and has been reported to play context-dependent functions. Its presence in follicular fluid is associated with high-quality embryo development in intracytoplasmatic injection patients [53], suggesting a positive role in ovarian function and oocyte quality. However, in polycystic ovary syndrome patients, miR-197-3p is significantly downregulated in GC and ovarian tissues. Functional studies suggest that its overexpression induces apoptosis by modulating key apoptotic proteins such as caspase 3, Bax, and Bcl-2 [54]. In contrast, its overexpression has been shown to attenuate apoptosis, inflammation, and oxidative stress in bronchial epithelial cells within a chronic obstructive pulmonary disease model [55].

Other miRNAs have also been determined in our analysis with potential roles in reproductive processes. For example, miR-2387 is expressed in sheep skeletal muscle during embryogenesis, though its function remains to be elucidated [56]. Similarly, miR-2285av was found to be upregulated in conditioned media from embryos that reach the blastocyst stage compared to those arrested at the 8–16-cell stage, suggesting a possible association with developmental competence [57]. Although their precise roles in ovarian or follicular physiology remain to be elucidated, their differential expression in our OS-EVs samples may indicate a broader involvement in early developmental processes or stress-responsive signaling.

## Conclusion

This study provides new insights into the response of bovine GCs to oxidative stress, highlighting the differential expression of miRNAs both within the cells and in their secreted EVs. Oxidative stress altered the miRNA cargo of GC-derived EVs, suggesting a coordinated regulatory response aimed at maintaining follicular function and homeostasis. Notably, several EVs-associated miRNAs more abundant under stress conditions are known to modulate apoptosis, cell proliferation, and inflammatory pathways, supporting the hypothesis that EVs-miRNAs may serve as paracrine signals to mediate stress adaptation within the follicular microenvironment. The identification of ruminant-specific and stress-responsive miRNAs further highlights the potential of EVs-miRNAs to be used as diagnostic biomarkers in reproductive biotechnologies. Future functional studies are warranted to elucidate the specific roles of these miRNAs in maintaining follicular function and enhancing oocyte competence under oxidative stress.

## Supplementary Information

Below is the link to the electronic supplementary material.

Additional File 1.

Additional File 2.

Additional File 3.

Additional File 4.

Additional File 5.

Additional File 6.

Additional File 7.

Additional File 8.

Additional File 9.

Additional File 10.

## Data Availability

All data generated or analyzed during this study are included in this published article. MicroRNA sequencing data have been deposited in the NCBI Gene Expression Omnibus (GEO) under accession number GSE304292. Extracellular vesicles isolation and characterization data have been deposited in the EV-TRACK knowledgebase with the ID EV260005.

## Abbreviations

–OH: Hydroxyl radicals
AU: Arbitrary units
BP: Biological processes
CWCS: Cumulative weighted context score
DE: Differential expression
EVs: Extracellular vesicles
FBS -: Exosome depleted fetal bovine serum
FBS: Fetal bovine serum
FC: Flow cytometry
FDR: False discovery rate
FDW: 0.1 µm filtered distilled water
FPBS: 0.1 µm filtered phosphate-buffered saline
FSC: Forward scatter
GCs: Granulosa cells
GEO: Gene expression omnibus
GFP: Green fluorescence protein
GO: Gene ontology
H_2_DCFDA: 2′, 7′-dichlorofluorescin diacetate
H_2_O_2_: Hydrogen peroxide
IntDen: Integrated density
IVC: In vitro culture
IVM: In vitro maturation
KEGG: Kyoto encyclopedia of genes and genomes
LFC: Log2 fold change
miRNAs: microRNAs
MWCO: Molecular weight cutoff
NGS: Next-generation sequencing
NTA: Nanoparticle tracking analysis
O2–: Superoxide anion
PBS: Phosphate-buffered saline
PBS−: Phosphate-buffered saline without calcium and magnesium
PCA: Principal component analysis
ROIs: Regions of interest
ROS: Reactive oxygen species
SD: Standard deviation
SEC: Size-exclusion chromatography
SEM: Standard error of the mean
SSC: Side scatter
TEM: Transmission electron microscopy
