# Supplementary figures and images for: Changes in the microRNA cargo of granulosa cell-derived extracellular vesicles under oxidative stress in a bovine model
Source: Biol Res. 2026 Mar 25;59:28. doi: 10.1186/s40659-026-00689-8 (PMC13137698; doi:10.1186/s40659-026-00689-8)

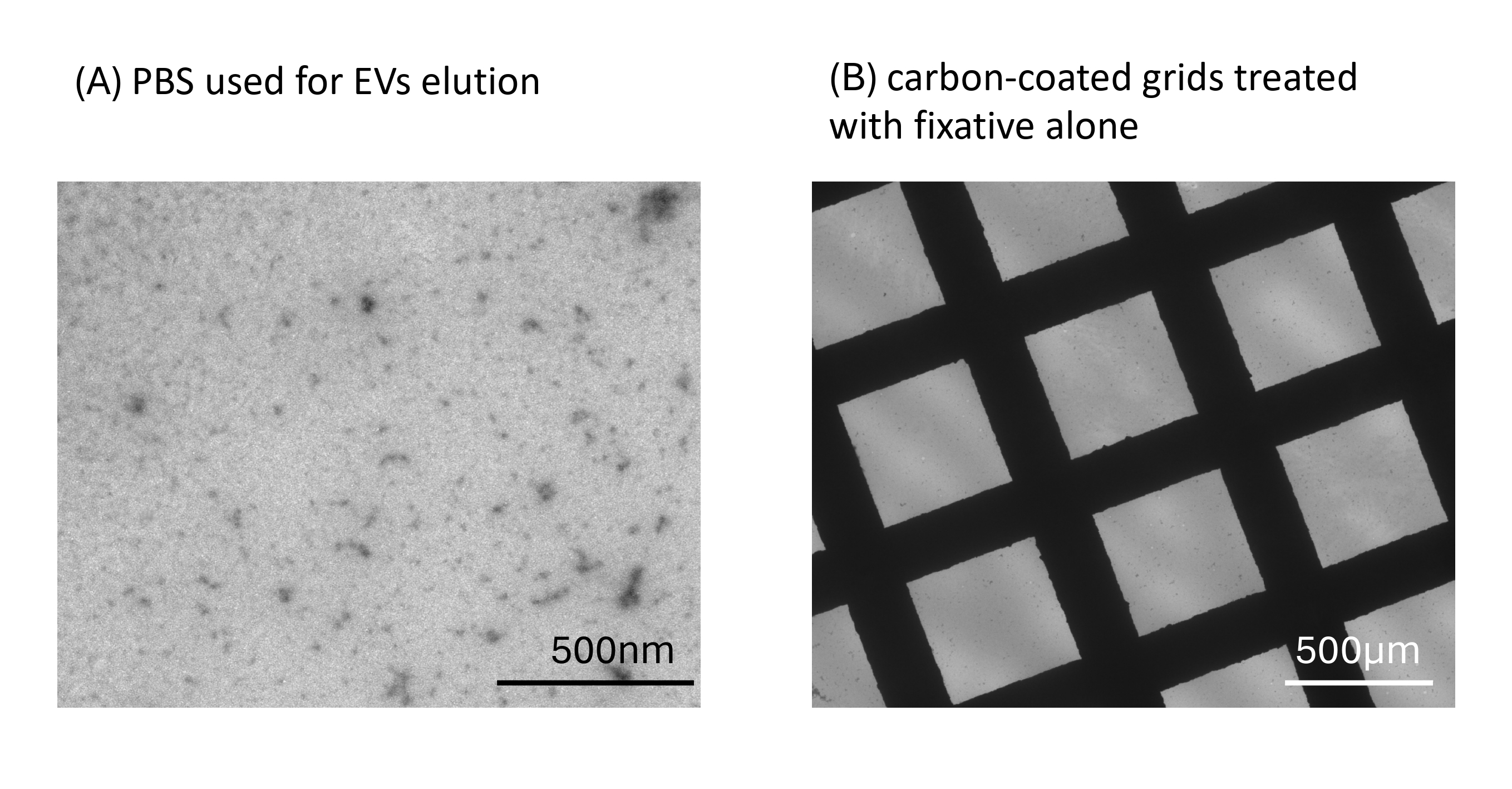

Supplement: Supplementary file 10 — Additional File 10. [file 40659_2026_689_MOESM10_ESM.png]
